# Commute Times, Food Retail Gaps, and Body Mass Index in North Carolina Counties

**Published:** 2010-08-15

**Authors:** Stephanie B. Jilcott, Haiyong Liu, Justin B. Moore, Jeffrey W. Bethel, James Wilson, Alice S. Ammerman

**Affiliations:** Department of Public Health, East Carolina University; East Carolina University, Greenville, North Carolina; East Carolina University, Greenville, North Carolina; East Carolina University, Greenville, North Carolina; Northern Illinois University, DeKalb, Illinois; University of North Carolina at Chapel Hill, Chapel Hill, North Carolina

## Abstract

**Introduction:**

The prevalence of obesity is higher in rural than in urban areas of the United States, for reasons that are not well understood. We examined correlations between percentage of rural residents, commute times, food retail gap per capita, and body mass index (BMI) among North Carolina residents.

**Methods:**

We used 2000 census data to determine each county's percentage of rural residents and 1990 and 2000 census data to obtain mean county-level commute times. We obtained county-level food retail gap per capita, defined as the difference between county-level food demand and county-level food sales in 2008, from the North Carolina Department of Commerce, and BMI data from the 2007 North Carolina Behavioral Risk Factor Surveillance System. To examine county-level associations between BMI and percentage of rural residents, commute times, and food retail gap per capita, we used Pearson correlation coefficients. To examine cross-sectional associations between individual-level BMI (n = 9,375) and county-level commute times and food retail gap per capita, we used multilevel regression models.

**Results:**

The percentage of rural residents was positively correlated with commute times, food retail gaps, and county-level BMI. Individual-level BMI was positively associated with county-level commute times and food retail gaps.

**Conclusions:**

Longer commute times and greater retail gaps may contribute to the rural obesity disparity. Future research should examine these relationships longitudinally and should test community-level obesity prevention strategies.

## Introduction

In the United States, the prevalence of obesity is higher in rural than in urban populations (1-5). Area-level factors that contribute to this disparity are not well understood, but one underlying mechanism may be the food environment. Obesity prevalence is lower in census tracts containing a supermarket (6), and rural areas have few supermarkets, which generally have a healthier mix of low-cost food items compared with local convenience stores (7). Accessibility to healthy food is also difficult in rural areas because convenience stores are more common than supermarkets (8-10).

Rural residents may regularly travel to urban areas in neighboring counties to shop for food because of convenience along the route to work, better prices, wider selection, or one-stop shopping offered at discount "supercenters" (eg, Walmart) (11,12). This pattern of food shopping among rural residents may create a retail shortfall or "gap" for food venues in rural areas, causing rural food venues to have a decreased share of the market. A large food retail gap may exacerbate rural food deserts (13), or areas where residents have limited access to affordable, healthy food (14), when smaller food venues in underserved areas close as business is lost to nearby discount supercenters (13,15). Rural residents' prolonged travel time to larger supermarkets or supercenters not only increases the retail gap in the rural county but decreases the frequency of food shopping. In turn, diet quality may decrease as rural residents purchase less fresh produce and more processed foods (16,17).

Another hypothesized mechanism underlying the rural-urban obesity disparity is that rural residents may spend more time traveling to work or to obtain goods and services than do their urban counterparts. Obesity is associated with urban sprawl (18-20), time spent in cars (21), and vehicle miles traveled per day (22). One Los Angeles-based study found that distance traveled to the nearest supermarket was positively associated with higher body mass index (BMI) (23). To our knowledge, no studies have examined the associations between distance to food shopping location, commute times, and BMI among rural and urban residents.

To better understand associations between area-level factors and obesity, we conducted ecologic analyses of associations between the percentage of rural-dwelling residents, commute times, food retail gaps, and BMI for all 100 North Carolina counties. We hypothesized that 1) the percentage of rural residents per county is positively correlated with commute time and food retail gap per capita, 2) county-level commute time is positively correlated with food retail gap per capita, and 3) both commute time and food retail gap per capita are positively correlated with county-level mean BMI. In separate individual-level, contextual analyses, we examined individual-level BMI as the dependent variable and county-level commute time and food retail gap per capita as independent variables. We hypothesized that longer commute times and greater food retail gaps per capita would be positively correlated with individual-level BMI.

## Methods

### Percentage of rural residents

We calculated the percentage of rural residents for all North Carolina counties by dividing the number of county residents who lived in a rural area according to 2000 census criteria (24) by the county population. The percentage of rural residents ranged from 4% to 100%.

### Commute times

We generated reports for county-level commute times for 1990 and 2000 from US census data from the North Carolina Department of Commerce Economic Development Intelligence System. Census data were derived from answers to the census long-form questionnaire. Respondents who worked outside the home estimated the number of minutes it took to get from home to work each day, and commute time was derived by dividing the total number of minutes by the number of workers aged 16 years or older who did not work at home. We examined associations by using the 1990 and 2000 commute times and the difference in commute times between 1990 and 2000. The difference in 1990 and 2000 commute times describes broad shifts in county-level commuting over 10 years.

### Food retail gap

We defined the food retail gap as the difference between county-level demand for food and county-level sales of food. We obtained the food retail gap for each North Carolina county from the North Carolina Department of Commerce Economic Development Intelligence System. The Environmental Systems Research Institute (ESRI) calculated retail gaps by subtracting county-level retail sales (supply) of products for a particular industry category in 2008 from county-level demand for products in that industry category in 2008. ESRI estimated demand using data on consumer expenditures from the Bureau of Labor Statistics and InfoUSA, a commercial database marketing system.

ESRI calculates the food retail gap for North American Industry Classification System (NAICS) codes 445 (representing food and beverage stores) and 722 (representing the food services and drinking places) separately. For these analyses, we used food retail gaps calculated from individual and combined NAICS codes 445 and 722. Venues included in the food and beverage stores subsector (NAICS code 445) sell food and beverages from fixed point-of-sale locations, such as supermarkets, grocery stores, convenience stores, meat markets, produce markets, and specialty food stores. Venues included in the food services and drinking places subsector (NAICS code 722) prepare meals, snacks, and beverages to customer order for consumption on and off the premises, such as full-service restaurants, limited-service eating places (fast-food restaurants), special food services, and drinking places. To control for population density, we calculated the food retail gap per capita by dividing the ESRI-estimated food retail gap by the 2007 county population estimate provided by the US census. A negative retail gap indicated that county-level sales were greater than county-level demand; a positive retail gap indicated that county demand was greater than county sales. For example, if County X has 1 chain supermarket and neighboring County Y has a large discount supercenter, residents of County X may begin grocery shopping at the supercenter, creating a positive food retail gap in County X and a negative food retail gap in County Y as residents' food dollars are spent in the neighboring county. This could result in closing of the 1 chain supermarket in County X, making travel to the discount supercenter a necessity for obtaining groceries.

### Body mass index

We estimated county-level mean BMI using self-reported height and weight for respondents to the North Carolina Behavioral Risk Factor Surveillance System (BRFSS); responses were aggregated over 5 years (2003-2007). The 5-year aggregate provided an adequate number of responses for reliable estimates for counties with low population densities (single-year estimates for rural counties are unstable). We calculated mean weighted BMI using SUDAAN version 10.1 (Research Triangle Institute, Research Triangle Park, North Carolina), which accounts for BRFSS oversampling of minorities. The mean (standard deviation) county-level BMI was 27.7 (0.85) kg/m^2^. The median (interquartile range) was 27.6 (25.9-30.1) kg/m^2^.

We conducted individual-level, contextual analyses using data from the 2007 North Carolina BRFSS for respondents aged 18 to 65 years with valid county identifiers. Because of confidentiality concerns, BRFSS does not provide county identifiers for residents of counties with fewer than 50 respondents. We excluded those counties. The individual-level sample consisted of 9,375 respondents from 64 counties. The mean population of the 64 counties included was 123,968, and the mean population of the 36 counties excluded was 25,322. The individual-level mean (SD) BMI was 28.1 (6.4) kg/m^2^.

### County-level census data

To control for economic interdependence of adjacent counties, we examined the Rural to Urban Continuum Codes (RUCC) as a covariate. The RUCC is a 9-level ordinal scale used by the Economic Research Service to classify counties according to adjacency to metropolitan areas (24). We included a diversity index as a potential covariate in analyses because of associations between racial/ethnic mix and availability of food venues (eg, supermarkets [25], fast-food restaurants [26]) and to account for North Carolina counties' varied race/ethnicity distributions (27). The diversity index represents the percentage of times 2 randomly selected people in each county would differ by race/ethnicity (27). The index is calculated by squaring the proportions of residents in each racial/ethnic group, summing the squares, and subtracting the result from 1. We determined both the county-level diversity index and the percentage of residents who lived below the poverty level using 2000 census data. We calculated the percentage of residents who lived below the poverty level by dividing the number of residents below the poverty level in 1999 by the estimated 1999 county population. North Carolina is divided into 3 regions (Coastal Plain/Eastern, Appalachian Mountain/Western, and Piedmont Plateau) with distinct demographic and socioeconomic characteristics. Thus, we also examined the variable "region" as a potential covariate.

### Statistical analyses

For county-level ecological analyses, we used SAS version 9.2 (SAS Institute, Inc, Cary, North Carolina) to calculate correlation coefficients for percentage of rural residents, food retail gap per capita, commute time, and BMI for all 100 North Carolina counties. We used backward selection to construct linear regression models to examine the associations among county-level independent variables of commute times and food retail gap per capita, using county-level mean BMI as the dependent variable. Percentage of rural residents, diversity index, percentage below poverty, and region were potential covariates and were eliminated from the model in successive steps if the *P* value for the parameter estimate was .05 or higher. We examined the potential multicollinearity among covariates by computing their corresponding tolerance values. The tolerance is the proportion of variance in a given independent variable that is not explained by all of the other covariates; we found a tolerance value for all of greater than 0.1, which has been widely used as the threshold for multicollinearity in linear regressions (28).

For individual-level, contextual analyses, we constructed multilevel linear regression models; the dependent variable was individual-level BMI from 2007 BRFSS respondents (n = 9,375). County-level independent variables were food retail gap per capita, commute time in 2000, and difference in commute times between 2000 and 1990. Sex, age, race/ethnicity, and education were individual-level covariates, and the RUCC was a county-level covariate. Region was added as a third level.

Multilevel regression analyses allowed us to assess associations between individual-level BMI and area-level factors, accounting for the fact that people who reside in the same county are not independent observations (29). We examined the association between individual-level BMI and the 5 county-level variables of interest (commute time in 2000, difference between 1990 and 2000 commute times, retail gap per capita for NAICS code 445 [food and beverage stores], retail gap per capita for NAICS code 722 [restaurants and drinking places], and combined retail gap per capita) in separate models. The first 3 models to examine the association between BMI and county-level variables of interest were 2-level random intercept models. Model 4 included additional regional dummy variables to account for fixed effects from region. We used SAS version 9.2 for individual-level, contextual analyses, with estimates weighted to adjust for BRFSS oversampling.

## Results

Summary statistics for the individual-level data among 2007 BRFSS respondents by region are reported in Table 1.

### County-level analyses

Percentage of rural residents was significantly correlated with both the commute times in 1990 and 2000 and the difference in commute times between the 2 years, food retail gap per capita for restaurants and drinking places, overall food retail gap per capita, and BMI (Table 2).

We found significant positive correlations between commute time and retail gap per capita  (Table 3). There were significant positive correlations between total food retail gap per capita and BMI and between the difference in commute times from 1990 to 2000 and BMI.

In linear regression analyses adjusted for county-level diversity index and the percentage of residents below the poverty level, a positive association was found between commute time in 2000 and BMI (parameter estimate, 5.24; standard error, 1.86; *P* = .006). We also found a significant positive association between food retail gap per capita and BMI when controlling for region and population percentage below poverty (parameter estimate, 0.024; standard error, 0.006; *P* < .001).

In linear regression models with county-level mean BMI as the dependent variable and difference in commute times from 1990 to 2000 and retail gap per capita as independent variables, the most parsimonious model included the covariates population percentage below poverty and regional fixed effects and explained 43% of variance in county-level BMI. When 2000 commute time and food retail gap per capita were included as independent variables, controlling for diversity index and percentage below the poverty level, the model explained 40% of variance in county-level BMI.

### Individual-level analyses

The point estimates for each of the county-level variables of interest (commute time and retail gap per capita) are presented for 4 model specifications (Table 4). In Model 1, we did not include any additional covariates. Individual covariates were added in models 2 and 3. In model 4, regional fixed effects were added. All 5 measures of county-level commute time and food retail gap per capita were positively associated with individual-level BMI. These effects were significant in the unadjusted model (model 1), and the significance remained when individual-level and regional covariates were included in models 2, 3, and 4, with the exception of average commute time increase in model 4. When 2000 commute time and retail gap per capita were both included in the same model with individual-level and regional covariates, the parameter estimates for the county-level variables of interest were no longer significant.

## Discussion

Our results demonstrate a positive correlation between percentage of rural residents and 1) commute times and 2) food retail gap per capita, suggesting that counties with a higher percentage of rural residents have longer commute times and greater retail shortfalls, and thus residents may generally spend food dollars outside their county of residence. Previous studies have found positive associations between BMI and travel distance to grocery stores (23) and time spent in cars (21,22).

We found significant cross-sectional correlations between individual-level and county-level BMI and 1) commute times and 2) food retail gap per capita, but significance did not remain when both were included in the individual-level model. This attenuation could be due to model over-adjustment if commute time and retail gap are both on the causal pathway explaining the relationship between rural residence and BMI.

These analyses support strategies presented in *Recommended Community Strategies and Measurements to Prevent Obesity in the United States* (30) to improve geographic availability of supermarkets in underserved areas and provide incentives to food retailers to offer healthier food and beverage choices in underserved areas. If implemented, these strategies would decrease travel times necessary for accessing healthy, affordable foods among low-income and rural residents. When combined with health education efforts and mass media campaigns encouraging healthy food choices, more accessible and affordable healthy foods may lead to healthier food consumption patterns and to lower obesity prevalence in these groups.

In a qualitative study of rural Georgia adults, participants identified several barriers to obtaining healthy foods, including poor selection, limited time, fuel prices, and the distance (15-45 miles) to larger communities with bigger stores and better selection (31). Another study found that longer distance traveled to the primary grocery store was associated with higher BMI (23). This previous work, taken together with our results, supports the notion that rural residents who travel farther to shop for food may purchase less healthful food. However, we did not measure the distance to the locations where people shopped and assumed that a positive food retail gap indicated a general trend for rural residents to shop for food outside their county of residence. Future work should assess the relationship between commute times and the locations where they purchase food. Future work should also include mediational analyses to examine the relationships between commute time, food shopping frequency and location, diet quality, and BMI.

This study has several limitations. Foremost is the ecological design, which used several different data sources. The inconsistent timing of data collection for commute times (1990, 2000), food retail gaps (2008), and BMI (2003-2007) is an additional limitation. However, we used the most recent data available, and average commute time is a proxy for distance between place of employment and residence (32). A related limitation is the exclusion of people in the 36 counties where BRFSS did not provide county-level identifiers, pointing to the need for more work to examine rural populations. An additional caveat is that we used self-reported height and weight from BRFSS to calculate BMI, potentially biasing results toward the null if hypothesized relationships between commute times, food retail gaps, and BMI truly exist, because of potential underestimation of weight status. The use of a commercial business database (InfoUSA) to obtain sales data is also a limitation, because such databases may contain errors (33). Finally, in these analyses, we assumed commute time referred to time spent driving. Some people may walk or bike to work instead of drive; however, few Americans actively commute (34).

This study is the first to examine correlations between commute times, food retail gap per capita, and mean BMI in counties in North Carolina. We present an approach to studying the association between BMI and variables related to the built and economic environments, providing support for the notion that economic and built environment factors are related to obesity.

## Figures and Tables

**Table 1:** Characteristics of 9,375 Respondents by Region, North Carolina Behavioral Risk Factor Surveillance System, 2007^a^

Characteristic	Region^b^		

	Western n = 1,789	Eastern n = 3,190	Piedmont n = 4,837
BMI^c^, kg/m^2 ^	27.8 (6.1)	28.7 (6.6)	27.9 (6.3)
Age, y	47.6 (12.1)	46.3 (12.4)	46.0 (11.9)
Men, %	37.2 (48.3)	35.7 (47.9)	37.0 (48.3)
High school diploma, %	58.6 (49.3)	60.5 (48.9)	50.5 (50.0)
Non-Hispanic black, %	5.0 (21.8)	24.8 (43.2)	18.0 (38.4)
Non-Hispanic white, %	87.9 (32.6)	63.4 (48.2)	72.3 (44.7)
Hispanic, %	3.8 (19.1)	5.3 (22.5)	5.4 (22.5)
County-level percentage residing in rural areas	57.9 (22.6)	44.8 (24.2)	25.8 (20.9)
County-level diversity index × 100^d^	18.6 (7.1)	49.0 (12.1)	43.6 (10.3)
County-level percentage below the poverty level	12.1 (2.0)	15.5 (3.8)	9.8 (1.8)
County-level commute time in 1990, minutes	19.4 (1.4)	19.5 (2.3)	19.9 (1.7)
County-level commute time in 2000, minutes	22.5 (2.0)	24.0 (3.3)	24.0 (2.2)
Commute time difference (2000 − 1990), minutes	3.2 (1.2)	4.4 (1.6)	4.1 (0.8)
Retail gap per capita (NAICS code 445)^e^	−251.2 (−353.3 to 124.2)	63.9 (−120.9 to 159.4)	−44.3 (−279.7 to 373.9)
Retail gap per capita (NAICS code 722)^f^	150.7 (−362.2 to 228.6)	−116.5 (−211.5 to 358.1)	98.8 (−152.8 to 361.9)
Combined retail gap per capita (NAICS codes 445 + 722)	−6.5 (−668.8 to 334.4)	−147.6 (−455.7 to 457.9)	240.4 (−85.9 to 517.4)

Abbreviations: BMI, body mass index; NAICS, North American Industry Classification System.

a Respondents resided in 64 North Carolina counties with valid values for all covariates for regression analyses, weighted to population.

b All values are reported as mean (standard deviation), except those for retail gap per capita, which are reported as median (interquartile range)

c BMI was unavailable for 441 respondents: 73 for the Western region, 143 for the Eastern region, and 225 for the Piedmont region.

d Calculated by squaring the proportions of residents in each racial/ethnic group, summing the squares, and subtracting the result from 1 (27)

e Retail gap per capita calculated by subtracting county-level sales of products for a NAICS category in 2008 from county-level demand for products in that category in 2008. NAICS code 445 defined as stores that sell food and beverages from fixed point-of-sale locations, including supermarkets, grocery stores, convenience stores, meat markets, produce markets, and specialty food stores

f NAICS code 722 defined as food services and drinking places that prepare meals, snacks, and beverages to customer order for consumption on and off the premises, including full-service restaurants, limited-service eating places (fast-food restaurants), special food services, and drinking places.

**Table 2:** Correlation Between Percentage of Rural Residents in 100 North Carolina Counties and Mean Commute Times, Food Retail Gap Per Capita, and BMI

**Variable**	Correlation With Percentage of Rural Residents^a^
Commute time 1990	0.56
Commute time 2000	0.59
Commute time difference (2000 − 1990)	0.25
Retail gap per capita (NAICS code 445)^b^	0.19
Retail gap per capita (NAICS code 722)^c^	0.43
Combined retail gap per capita (NAICS codes 445 + 722)	0.31
County-level BMI	0.21

Abbreviations: BMI, body mass index; NAICS, North American Industry Classification System.

a *P* values ranged from <.001 to .04 using a *t* test except that for retail gap per capita (NAICS code 445) (*P* = .06).

b Retail gap per capita calculated by subtracting county-level sales of products for a NAICS category in 2008 from county-level demand for products in that category in 2008. NAICS code 445 defined as stores that sell food and beverages from fixed point-of-sale locations, including supermarkets, grocery stores, convenience stores, meat markets, produce markets, and specialty food stores.

c NAICS code 722 defined as food services and drinking places that prepare meals, snacks, and beverages to customer order for consumption on and off the premises, including full-service restaurants, limited-service eating places (fast-food restaurants), special food services, and drinking places.

**Table 3:** Correlation Between BMI and Mean Commute Times and Food Retail Gap per Capita in 100 North Carolina Counties

Variable	Retail Gap per Capita^a^			

	NAICS Code 445^b^	NAICS Code 722^c^	NAICS Codes 445 + 722	County-Level BMI
Commute time 1990	0.26	0.41	0.34	0.12
Commute time 2000	0.35	0.51	0.44	0.31
Commute time difference (2000 − 1990)	0.29	0.35	0.34	0.46
County-level BMI	0.37	0.24	0.34	1.00

Abbreviations: BMI, body mass index; NAICS, North American Industry Classification System.

a Retail gap per capita calculated by subtracting county-level sales of products for a NAICS category in 2008 from county-level demand for products in that category in 2008. *P* values ranged from <.001 to .01 using a *t* test except that for commute time in 1990 and BMI (*P* = .22).

b NAICS code 445 defined as stores that sell food and beverages from fixed point-of-sale locations, including supermarkets, grocery stores, convenience stores, meat markets, produce markets, and specialty food stores.

c NAICS code 722 defined as food services and drinking places that prepare meals, snacks, and beverages to customer order for consumption on and off the premises, including full-service restaurants, limited-service eating places (fast-food restaurants), special food services, and drinking places.

**Table 4:** Correlation Between Individual-Level BMI and County-Level Variables, North Carolina^a^

County-Level Variable	Regression Model^b^,^c^			

	1	2	3	4
2000 Commute time	0.0847	0.0730	0.0733	0.0660
Commute time difference (2000 – 1990)	0.2719	0.1812	0.1791	0.1572
Retail gap per capita (NAICS code 445)^d^	0.0004	0.0002	0.0003	0.0002
Retail gap per capita (NAICS code 722)^e^	0.0004	0.0004	0.0004	0.0004
Combined retail gap per capita (NAICS codes 445 + 722)	0.0002	0.0002	0.0002	0.0002

Abbreviations: BMI, body mass index; NAICS, North American Industry Classification System.

a Individual-level BMI was the dependent variable and county-level commute times and food retail gap per capita were independent variables. Individual covariates were age, age squared, sex, education, and race/ethnicity

b Model 1: no additional covariates; model 2: individual covariates only; model 3: individual covariates + Rural to Urban Continuum Codes (RUCC) (24); model 4: individual covariates + RUCC + regional dummy variables.

c *P* values ranged from <.001 to .048 using a *t* test, except those for Model 2 for retail gap per capita (NAICS code 445 [*P* = .08] and NAICS code 722 [*P* = .06]) and for Model 4 for retail gap per capita (NAICS code 445 [*P* = .06]).

d Retail gap per capita calculated by subtracting county-level sales of products for a NAICS category in 2008 from county-level demand for products in that category in 2008. NAICS code 445 defined as stores that sell food and beverages from fixed point-of-sale locations, including supermarkets, grocery stores, convenience stores, meat markets, produce markets, and specialty food stores.

e NAICS code 722 defined as food services and drinking places that prepare meals, snacks, and beverages to customer order for consumption on and off the premises, including full-service restaurants, limited-service eating places (fast-food restaurants), special food services, and drinking places.
